# Preclinical testing of miRNA-193b-3p mimic in acute myeloid leukemias

**DOI:** 10.1038/s41375-023-01937-6

**Published:** 2023-06-13

**Authors:** Hasan Issa, Raj Bhayadia, Robert Winkler, Laura Elise Swart, Dirk Heckl, Jan-Henning Klusmann

**Affiliations:** 1 Department of Pediatrics, Goethe-University Frankfurt, Frankfurt, Germany; 2 Frankfurt Cancer Institute, Goethe University Frankfurt, Frankfurt am Main, Germany; 3 German Cancer Consortium (DKTK), Partner Site Frankfurt/Mainz and German Cancer Research Center (DKFZ), Heidelberg, Germany; 4 Princess Maxima Centrum for Pediatric Oncology, Utrecht, The Netherlands; 5 Pediatric Hematology and Oncology, Martin-Luther-University Halle-Wittenberg, Halle, Germany

**Keywords:** Targeted therapies, Acute myeloid leukaemia

## To the Editor

Despite intensive multi-agent chemotherapy and hematopoietic stem cell transplantation, cure rates for patients with acute myeloid leukemia (AML) remain poor [1]. One of the greatest obstacles in treating AML is managing toxicity, particularly in terms of bone marrow regeneration and the restoration of normal hematopoiesis [2]. This highlights the need for developing novel, more leukemia-specific therapies. In this preclinical study, we assessed the therapeutic potential of restoring miRNA-193p-3p (miR-193b) functions using lipid nanoparticles (LNP)-encapsulated miR-193b mimic in patient-derived xenograft (PDX) models of pediatric AML.

miRNAs can both promote and inhibit leukemia progression and maintenance by interacting with proto-oncogenes and key signaling pathways [3]. During hematopoiesis, miR-193b acts as an endogenous tumor suppressor by targeting several members of the RAS-RAF-MEK-ERK cascade (MAPK/ERK), which regulates proliferation and cell cycle progression [4]. The MAPK/ERK cascade is activated during differentiation but is repressed in hematopoietic stem cells (HSCs) [5, 6]. In HSCs activation of the (THPO)-MPL-STAT5 signaling cascade upregulates miR-193b, which prevents HSCs exhaustion by limiting self-renewal and proliferation [4, 7]. Previously, we demonstrated that miR-193b is globally downregulated across a spectrum of genetically diverse pediatric and adult AMLs, and its lower expression is associated with poor prognosis [4]. Lentiviral restoration of miR-193b expression abrogated leukemic growth in vitro and in vivo. Therefore, we hypothesized that restoring miR-193b function can provide anti-leukemic activity while preserving normal HSCs.

**Fig. 1:**
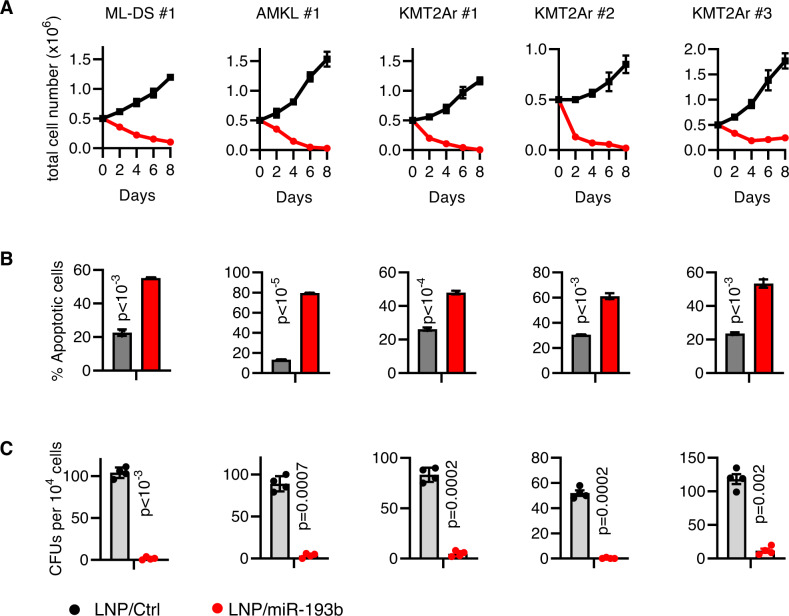
AML xenografts are susceptible to transient LNP/miR-193b treatment in vitro. **A** The absolute number of human AML PDX cells after treatment with single dose of 4 µg/ml LNPs. **B** Percentage of apoptotic cells (Annexin V^+^) on day 2 post treatment. **A**, **B** Data are represented as the mean ± standard deviation of three independent biological replicates (two-way ANOVA). **C** Absolute number of CFUs in methylcellulose-based colony-forming assays of LNPs-treated AML PDXs. Data are shown as the mean ± standard deviation of four plates from two biological replicates (two-way ANOVA).

To develop a clinically relevant approach for restoring miR-193b function, we encapsulated miR-193b mimics into Dlin-MC3-DMA nanoparticles (LNP/miR-193b) using a semi-automated platform that assures GMP-grade LNPs production, making it suitable for upscaling to clinical applications [8]. The loading efficiency of the mimics ranged from 75 to 85%, and the formulated LNPs exhibited a diameter of approximately 55 nm, a polydispersity index of 0.1, and a zeta potential between –2 and –3 mV (Supplementary Fig. 1A–C). On-target activity and anti-leukemic efficacy of LNP/miR-193b were verified in vitro using genetically well-characterized AML blasts derived from seven patients representing high-risk AML – including KMT2Ar and AMKL – and favorable-risk myeloid leukemia of Down syndrome (ML-DS) (Supplementary Table 1). The AML blasts showed significantly lower miR-193b expression (Supplementary Fig. 1D) than normal CD34^+^CD38^−^ or CD34^+^CD38^+^ hematopoietic stem and progenitor cells. In line with our previous findings [4], treatment with 4 µg/ml LNP/miR-193b showed strong anti-proliferative and pro-apoptotic activity in AML samples (Fig. 1A, B, Supplementary Fig. 1E, F). LNP/miR-193b treatment impeded colony formation by over 10-fold compared to the negative control nanoparticles, LNP/Ctrl (Fig. 1C, Supplementary Fig. 1G). MiR-193b is a negative regulator of c-KIT [4] and LNP/miR-193b treatment reduced c-KIT expression in AML PDXs (Supplementary Fig. 1H), indicating its on-target activity.

**Fig. 2:**
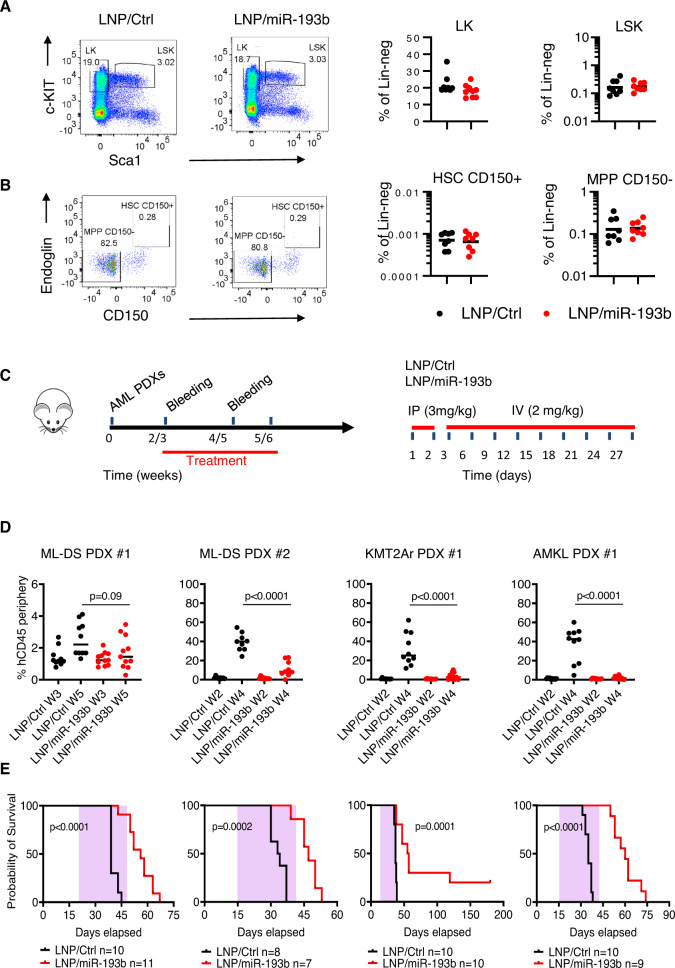
LNP/miR-193b treatment in vivo spares HSCs and delays leukemia propagation. Representative flow cytometry plots (left) and percentages (right) of murine LK and LSK cells following two weeks of LNPs treatment. **B** Representative flow cytometry plots (left) and percentages (right) of murine HSCs (CD150^+^) and MPPs (CD150^−^) after LNPs treatment. In **A** and **B**, lines represent the mean, n = 8 mice per arm. **C** Left, schematic illustration of LNP treatment for AML PDXs in vivo. Humanized immunodeficient mice were injected with leukemic cells on day 0 and treated with LNPs as shown in the treatment scheme on the right. **D** Percentage of human CD45^+^ cells in the periphery of transplanted mice. Blood sampling was performed one day before the start of the treatment and after two weeks. Lines represent the mean and statistical significance was calculated based on one-way ANOVA. **E** Kaplan-Meier survival curves of LNPs-treated PDXs. Statistical significance was calculated using the log-rank test.

Therapeutic interventions must eradicate leukemia without affecting healthy stem cells. Therefore, we investigated the safety of restoring miR-193b function by determining its effect on naive hematopoiesis. We treated immunocompetent C57B/6 J mice with LNPs encapsulating either murine miR-193b or negative control mimics (Supplementary Fig. 2A). We then characterized hematopoietic populations using multicolor flow cytometry [9]. We followed an optimized in vivo dose regimen, which assures RNAi-mediated knockdown of leukemic fusion oncogenes and thus therapeutic efficacy [8, 10]. The treatment regimen ensures liver saturation with LNPs and enrichment of the periphery and bone marrow with the nanoparticles. During the treatment period, mice did not show any reduction in activity or body weight (Supplementary Fig. 2B). LNPs treatment did not cause significant changes to distinct blood lineages. Gr1^+^CD11b^+^ myeloid-derived suppressor cells, B220^+^ B-cells, or CD3e^+^ T-cells (Supplementary Fig. 2C–E) percentages in the bone marrow were comparable between control and miR-193b treated mice. CD71^+^Ter119^+^ erythroid progenitors were marginally reduced in the miR-193b arm (Supplementary Fig. 2F) but the difference did not reach statistical significance. A detailed lineage-tracking analysis of Lin^−^Sca1^+^c-Kit^+^ (LSK) cells and their progenies revealed that transient restoration of miR-193b function also spared the hematopoietic stem and progenitor populations (Fig. 2A). The Lin^−^Sca1^−^cKit^+^ (LK) cell population presented with slightly lower expression of c-Kit in the LNP/miR-193b arm than in the control arm, but the difference was not statistically significant (Fig. 2A). No differences were observed in the long-term multilineage stem cell compartment (CD150^+^ HSC) or lineage-primed multipotent progenitors (CD150^−^ MPP, Fig. 2B). Furthermore, the oligopotent and lineage-primed progenitors of the megakaryocyte (MKp, pre-Meg), myeloid (pre-GM, GMP), and erythroid (pre-CFU-E, CFU-E, pro-Ery) lineages were unaffected by LNP/miR-193b treatment (Supplementary Fig. 2G and Supplementary Fig. 3). These data indicate that transient restoration of miR-193 function is safe, does not affect normal HSCs and their progeny and that LNP/miR-193b could be therapeutically exploited to inhibit leukemia expansion in vivo.

Therefore, we assessed the LNPs anti-leukemic effect in vivo using four AML PDXs (Fig. 2C). After the confirmation of leukemia engraftment, we randomized each xenograft recipient into one of the two treatment arms. Leukemia propagation in control-treated xenografts was evidenced by a substantial increase in hCD45^+^ cells in the periphery. In contrast, LNP/miR-193b treatment halted leukemia progression and decreased chimerism in the peripheral blood of recipient mice after two weeks of treatment (Fig. 2D). In three xenografts (ML-DS PDX #2, KMT2Ar PDX #1, and AMKL PDX #1), we observed a significant (*p* < 10^-4^) reduction in hCD45^+^ cells. LNPs treatment was well tolerated confirming the safety of restoring miR-193b function in immunocompromised hosts (Supplementary Fig. 4A, B). In all xenografts, LNP/miR-193b treatment doubled the median survival compared to control mice (Fig. 2E). In two mice, a short cycle of LNPs treatment resulted in a long-term survival without any signs of leukemia presence in the bone marrow. As leukemic engraftment was confirmed prior to treatment, these data imply that transient restoration of miR-193b function occurs in leukemia-initiating stem cells and can lead to the complete eradication of leukemia [10]. Future dose refinements, especially in combination with other drugs, may further enhance these anti-leukemic effects.

As expected, we did not observe differences in the percentage of leukemic blasts in the bone marrow of the recipient mice at the time of sacrifice (data not shown). However, spleen size was significantly reduced in mice that received LNP/miR-193b treatment compared to that in control animals (Supplementary Fig. 4C).

Taken together, by leveraging PDXs, this study overcomes the limitations of cell lines and provides a physiological model of clonal diversity in AML to test the functionality of miR-193b-based therapeutics. Our work supports not only the robust tumor-suppressive functionality of miR-193b but also the utility of miR-mimics for cancer treatment.

Interfering with miRNA expression using synthetic oligonucleotides has emerged as a promising treatment approach for leukemia and other malignancies [11]. While the inhibition of overexpressed oncogenic miRNAs can be achieved using antagomiRNAs and miRNA sponges, the restoration of miRNA function using mimics has proven challenging. The first-in-human phase I/II clinical trial of a liposomal miR-34a mimic (MIRX34) aimed to compensate for the reduced levels of miR-34a that frequently co-occur with TP53 loss and are linked to reduced survival in patients with solid tumors [11]. Although the trial was terminated early owing to immune-mediated toxicity and later-occurring adverse events, it was a proof-of-concept for miRNA-centered therapeutics [11]. Endogenous miR-34 controls the expression of over 30 proto-oncogenes that are crucial for the proliferation and invasion of malignant cells [12]. Similarly, retrieving miR-193b function can potentially affect several pathways, including the MAPK/ERK pathway, which is constitutively active in leukemia [4], and the well-known oncogene c-KIT, which is recurrently mutated in AML and is linked to therapy resistance, especially following relapse. Similar LNPs formulations encapsulating miR-193a-3p mimics (INT-1B3) are currently undergoing clinical trials for advanced solid tumors [13]. Both miR-193b and miR-193a have tumor suppressive activity and are downregulated in malignant tissues [4, 13].

Treatment with LNP/miR-193b was well tolerated and did not cause detectable changes in the HSCs. Mild differences observed in the hematopoietic progenitors and in differentiation of lymphoid and myeloid cells were expected given that miR-193b exhibits divergent cellular functions in different cell contexts. Multiple miR-193b targets repressed in HSCs are activated upon hematopoietic differentiation, specifically in the MAPK/ERK cascade [5, 7]. The cytokine-induced MAPKs play crucial roles in the regulation of differentiation, apoptosis and leukemia proliferation, but not in the maintenance of HSCs self-renewal [14, 15]. Thus, our LNPs treatment walked the fine line of retrieving miR-193b expression and its associated anti-leukemic activity without triggering HSCs exhaustion.

In conclusion, our data confirmed that the transient restoration of miR-193b has strong anti-leukemic effects that can be leveraged to treat leukemia in a clinically relevant system based on PDXs. Considering that aberrantly activated MAPK signaling is involved in a plethora of hematological diseases, and that mutations in several miR-193b targets are associated with poor clinical outcomes, our findings suggest that the clinical use of LNP/miR-193b may improve the efficacy and/or tolerability of current chemotherapeutic approaches.

## Supplementary information

SUPPLEMENTAL MATERIAL

## Data Availability

All the data are available in the article and supplementary files. Raw data files are available upon request from the corresponding author.
