# Supplementary material for: Preclinical testing of miRNA-193b-3p mimic in acute myeloid leukemias
Source: Leukemia. 2023 Jun 13;37(7):1583–7. doi: 10.1038/s41375-023-01937-6 (PMC10317836; doi:10.1038/s41375-023-01937-6)
Supplement: Supplementary file 1 — SUPPLEMENTAL MATERIAL [file 41375_2023_1937_MOESM1_ESM.docx]

# **Supplemental Material**

**Preclinical testing of miRNA-193b-3p mimic in acute myeloid leukemias**

Issa, Bhayadia et al.

# **Supplemental Methods**

**Patient samples**

Pediatric AML samples were collected from patients enrolled in the AML Berlin-Frankfurt-Münster treatment protocols for children and adolescents. Written informed consent was obtained from all patients and custodians in accordance with the Declaration of Helsinki and local laws and regulations. The study was approved by the institutional review boards of all participating centers. For details, see **Supplemental Table 1**.

**Measurement of miR-193b expression by qRT-PCR.** Total RNA was isolated from sorted CD34^+^CD38^+^, CD34^+^CD38^+^ hematopoietic stem and progenitor cells (HSPCs), and PDX cells using the miRNeasy Kit (#217084, Qiagen). Specific cDNA was synthesized for hsa-miR193b-3p (#002367, Thermo Fisher Scientific) and hsa-RNU24 (#001001, Thermo Fisher Scientific) using a high-capacity cDNA Reverse Transcription Kit (#4374966, Thermo Fisher Scientific). Quantitative real-time PCR (qRT-PCR) was performed on a StepOnePlus Real-Time PCR System (Thermo Fisher Scientific) using TaqMan Fast Advanced Master Mix (#4444556, Thermo Fisher Scientific).

**Lipid nanoparticle preparation and characterization.** The *in vivo* grade mirVana miRNA mimics (hsa-miR-193b-3p, #4464070, Thermo Fisher and mmu-miR-193b-3p, #4427975, Thermo Fisher) and the Negative Control (negative control #1, #4464061, Thermo Fisher) were hydrated in sodium acetate buffer (25 mM NaAc, pH 4) and quantified using the Qubit microRNA Assay (#Q32880, Thermo Fisher).

LNPs were generated by pumping 3 volumes of siRNA aqueous solution with 1 volume of lipid mixture through a microfluidic mixer (NanoAssemblr, Precision Nanosystems) at a combined flow rate of 4 ml/min. The final LNP/mimic solution was dialyzed overnight against PBS at 4°C. The mimic encapsulation efficiency was determined by lysing the LNPs with 0.1% TritonX for 15 min at 40°C and then quantifying the nucleic acids using the Qubit microRNA Assay. Malvern Zetasizer was used to determine the physical parameters. LNPs hydrodynamic diameter and polydispersity index were measured after diluting the mixture 1:100 in PBS and zeta potential measurement after applying 1:1000 dilution in dH_2_O.

**Cell culture**. AML PDX cells were cultured in StemSpan (#09650, StemCell Technologies) medium supplemented with 1% P/S, 50 ng/ml SCF, 20 ng/ml THPO, 50 ng/ml FLT3L, 5 ng/ml IL6, 2.5 ng/ml IL3, 1:250 lipid concentrate, 750 nM SR1 (# 72352, StemCell Technologies) and 35 nM UM171 (A15913, StemCell Technologies).

**LNP treatment.** 24 hours post thawing, cells were treated with 4 µg/ml LNPs at 1 × 10^6^ cells/ml for 24 h. **Proliferation assay,** 24 h post treatment, cells were diluted to 0.5 x 10^6^ cells/ml and counted every 48 h for 8 days. **Colony formation unit assays,** 5000 cells were taken 24 h post treatment, resuspended in 2.5 ml semisolid methocellulose-based media (#HSC003, R&D Systems) and 1 ml moved onto a 2 cm dish. Colonies were counted after seven days, with a cut-off of at least 25 cells per colony.

**Apoptosis assay.** Annexin V staining was performed 48 h after treatment. 0.1 x 10^6^ cells were washed once with PBS and stained with APC-conjugated Annexin V (#556547, BD Biosciences) according to the manufacturer's protocol.

**Flow Cytometry.** To determine c-KIT expression, 0.1 x 10^6^ cells were collected and washed once with FACS buffer (PBS, 5% FCS, 2 mM EDTA), stained with CD117 (#B49221, Beckman Coulter) and CD45-APC (#IM2473, Beckman Coulter) antibodies for 30 min at 4°C, washed once with FACS buffer, and resuspended in FACS buffer. Flow cytometry measurements were performed using CytoFLEX B4-R3-V5 (Beckman Coulter) and DAPI was used for live/dead staining. All assays were performed in triplicates.

**Animal work**

**LSK experiment**. 8-week-old C57BL/6J mice (Jackson Laboratory) were treated with 3 mg/kg of LNPs via an intraperitoneal (i.p.) route on days 1 and 2, and with 2 mg/kg intravenously (i.v.) on days 3, 6, 9, 12, and 14. Body weight was monitored during the treatment. LNPs were given by i.p. and i.v. routes based on the LNPs volume (i.v. up to 125 µl and i.p for volumes > 125 µl, based on 25 g body weight). Two days after the last LNPs dose, the mice were humanely culled, and cells were isolated from the bone marrow and processed for flow cytometry analysis, as described below.

**Survival experiments**. Six- to eight-week-old humanized immunodeficient mice [1] were sublethally irradiated with 2.5 Gy and transplanted with 1 × 10^6^ AML PDX cells. Leukemia engraftment was confirmed by tail bleeding prior to LNPs treatment, and the mice were randomized into two treatment groups.

The LNP treatment regimen consisted of two consecutive doses of 3 mg/kg i.p. on days 1 and 2, followed by nine doses of 2 mg/kg intravenously until day 27.

Body weight was monitored during treatment, and additional tail bleeding was induced after 2 weeks of treatment. Mice were humanely culled when they showed signs of sickness. Cells were harvested from the bone marrow and spleen, and leukemia burden was assessed by flow cytometry analysis.

To assess leukemia engraftment, 50 µL of blood was withdrawn from the tail vein of the transplanted mice, lysed with red blood cell lysis buffer, and stained with CD45-APC and CD33-PE (#A07775, Beckman Coulter).

Upon sacrifice, the engraftment of human cells was assessed in the bone marrow and spleen. The harvested cells were lysed in red cell lysis buffer and stained with CD45 and CD33. Flow cytometry measurements were performed using CytoFLEX B4-R3-V5 (Beckman Coulter) and DAPI was used for live/dead staining.

**Characterization of murine Lin^-^Sca1^+^c-Kit^+^ (LSK) and progenitor cells.** Bone marrow was harvested from LNP-treated C57BL/6J mice and erythrocytes were lysed with red blood cell lysis buffer. For lineage tracking of LSKs and immature progenitors, 5 x 10^6^ cells were stained with the following murine antibodies according to the panel developed by Pronk *et al.* [2]. Ter119-FITC (#561032, BD Biosciences), CD16/32-PerCp-Cy5.5 (#560540, BD Biosciences), CD150-PE-Cy7 (#115914, Biolegend), CD117-APC (#105812, Biolegend), CD41-APC-Cy7 (#133928, Biolegend), Lineage cocktail-Pacific Blue (#133310, Biolegend), CD71-BV605 (#563013, BD Biosciences), CD105-BV650 (#562759, BD Biosciences), and Sca1-BV711 (#744326, BD Biosciences). Flow cytometry measurements were performed using an LSR Fortessa (BD Biosciences) and DAPI was used for live/dead staining.

For the mature lineages, 0.5 x 10^6^ cells were stained with the following murine antibodies: CD71-FITC (#113806, Biolegend), CD41a-PE-Cy7 (#561424, BD Biosciences), CD42d-APC (#148506, Biolegend), Gr1-PerCp-Cy5.5 (#108428, Biolegend), CD11b-PE-Cy7 (#101216, Biolegend), CD3e-APC (#100312, Biolegend), B220-APC-Cy7 (#103224, Biolegend). Flow cytometry measurements were performed using CytoFLEX B4-R3-V5 (Beckman Coulter) and DAPI was used for live/dead staining.

**Statistical analysis**

Statistical analyses of experimental data were performed using GraphPad Prism 9, using unpaired Student’s t-tests or two-way ANOVA. The Kaplan-Meier method was used to generate survival curves, the log-rank test was used to evaluate survival differences, and quantitative data are presented as the mean ± SD.

**Ethical statement**

All animal experiments were performed according to protocols approved by the local authorities (Niedersächsisches Landesamt für Verbraucherschutz und Lebensmittelsicherheit and Landesverwaltungsamt Sachsen-Anhalt). Animals were maintained under pathogen-free conditions.

# **Supplemental Figures**


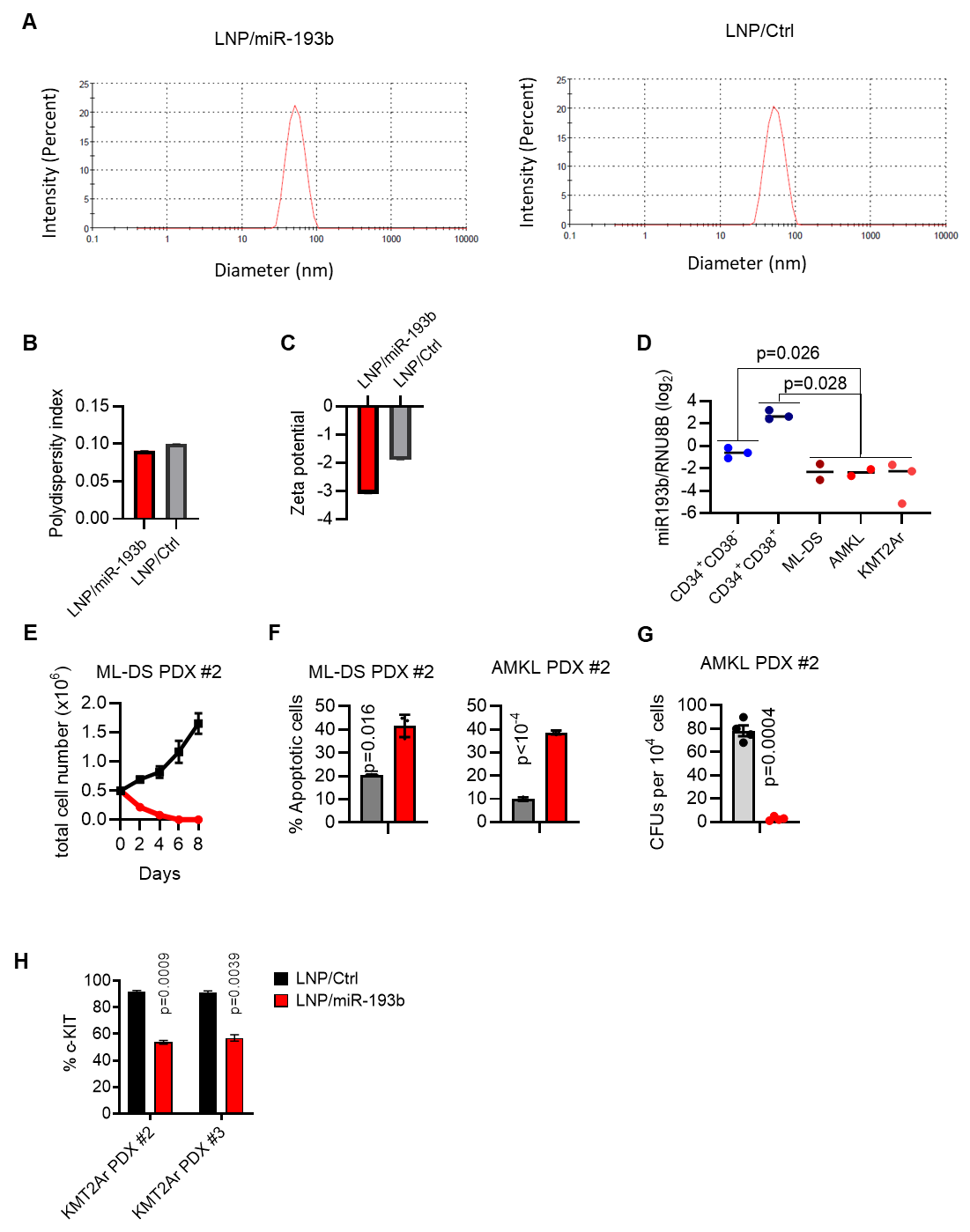


**Supplemental Figure 1.**

Physical parameters of formulated LNPs as measured in Zetasizer, (A) Size distributions of LNPs formulations, (B) polydispercity index and (C) Zeta potential. (D) Expression levels of miR-193b across AML PDX samples, CD34^+^CD38^-^ stem cells, and CD34^+^CD38^+^ immature progenitors from three independent donors were measured using TaqMan qRT-PCR (two-way ANOVA). (E) Absolute cell number of human PDX cells after treatment with LNPs. (F) Percentage of apoptotic cells (Annexin V^+^) on day 2. In E and F data are represented as mean ± standard deviations of 3 independent biological replicates (two-way ANOVA). (G) Absolute number of CFUs in methylcellulose-based colony forming assays of LNPs-treated AML PDXs. Data are shown as mean ± standard deviation of 4 plates from 2 biological replicates. (H) Percentages of c-KIT-expressing AML PDX cells after LNPs treatment. Flow cytometry analyses were performed on day 3. Data are shown as mean ± standard deviations of 3 independent biological replicates (two-way ANOVA).

**
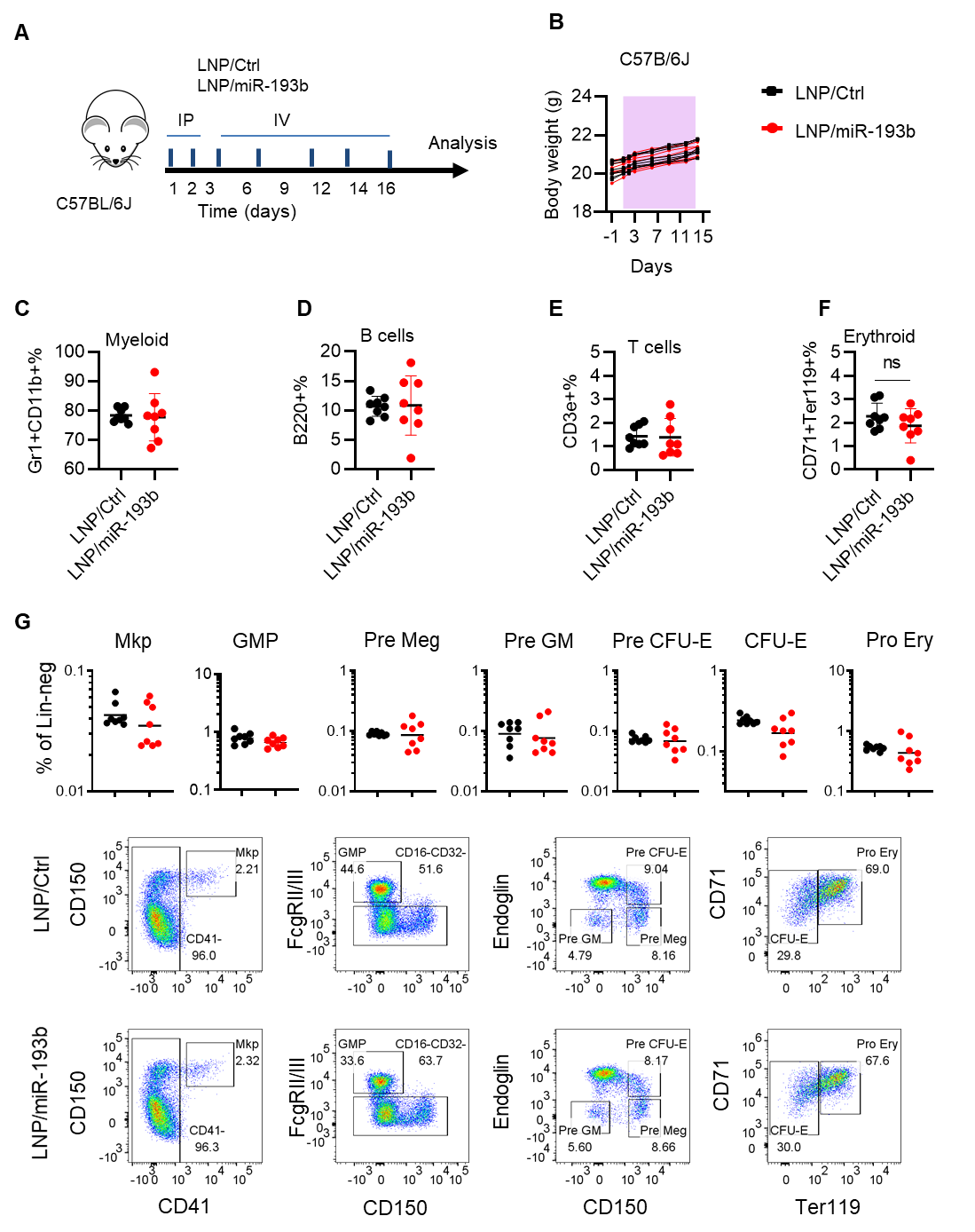
**

**Supplemental Figure 2.**

(A) Schematic illustration of LNPs treatment of C57BL/6J mice. Animals were treated with LNPs encapsulating either murine miR-193b mimics or negative control. (B) Weight of C57BL/6J mice during LNPs treatment. Each line represents a single mouse. Percentages of (C) myeloid cells, (D) B cells, (E) T cells and (F) erythroid progenitors in the bone marrow of C57BL/6J mice following LNPs treatment. (G) Representative flow cytometry plots (bottom) and percentages (top) of murine immature hematopoietic progenitors. The percentages of all populations were calculated from the lineage-negative fraction of gated single cells. In C-G, lines represent the mean, n=8 mice per arm and statistical significance was calculated based on one-way ANOVA.


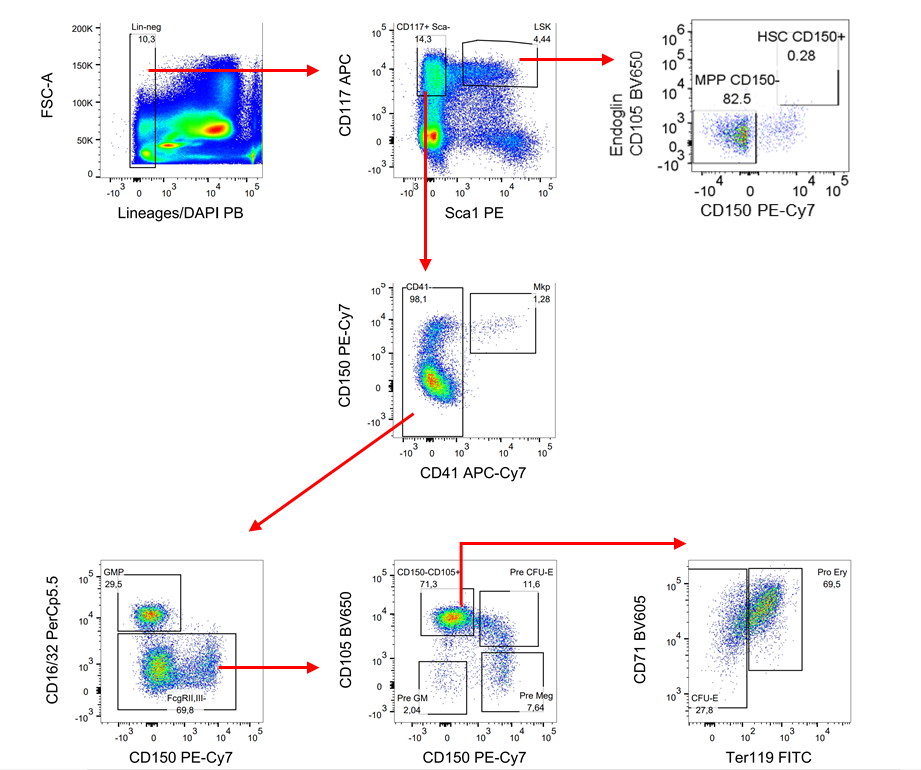


**Supplemental Figure 3.**

Gating strategy for characterization of murine hematopoiesis.

**
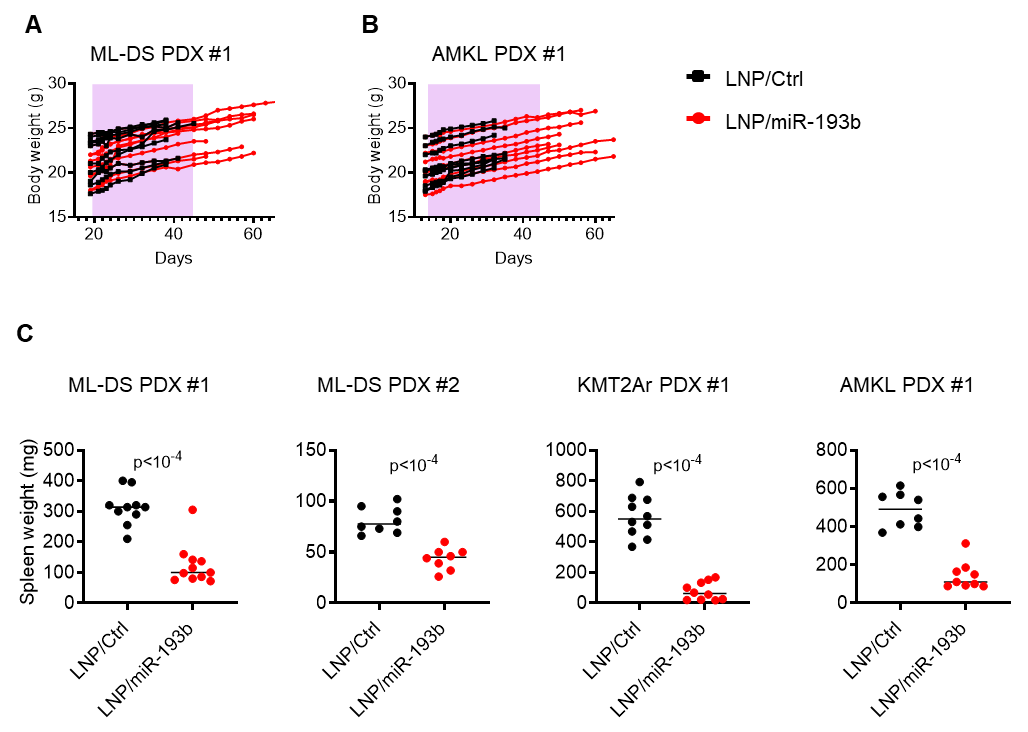
**

**Supplemental Figure 4.**

Weight of MISTRG mice transplanted with ML-DS PDX#1 (A) and AMKL PDX #1 (B) and treated with LNPs. Each line represents a single mouse. (C) Weight of spleens from mice treated with the LNPs. Lines represent the mean and statistical significance was calculated based on one-way ANOVA.

# **Supplemental Tables**

|  | gender | age at diagnosis (years) | WBC (x109/L) | hemoglobin (g/dl) | BM blasts (%) | CNS | SCT | molecular genetics | Cytogenetics (karyotype) | response | relapse |
| --- | --- | --- | --- | --- | --- | --- | --- | --- | --- | --- | --- |
| ML-DS PDX#1 | m | 1 1/3 | 4800 | 12 | 10.5 | no | no | GATA1 mutation | k.A. | CCR | no |
| ML-DS PDX#2 | m | 2 1/6 | 32500 | 7.9 | 70.5 | no | yes | GATA1 mutation | 47,XY,t(3;13)(q?26;q?13~14)  del(13)(q?14q22),+21c[cp14]/  47,sl,del?(15)(q?)[cp2]/46,XY[1] | NR,  CCR:after SCT | no |
| AMKL PDX#1 | m | 2/12 | 33800 | 9.5 | 34 | yes | no | n.d. | 46,XY,t(1;8;22)(p13;q22;q13)[14]/46,XY[1] | NR | no |
| AMKL PDX#2 | m | 1 | 40000 | 9.9 | 64 | no | yes | KMT2A mutation | 46,XY[15].nuc ish 3q26(EVI1x2)[100/100],  8q22(RUNX1T1x2),21q22(RUNX1x2)[98/100],  11 q23(MLLx2)[99/100], 16q22(CBFBx2)[100/100]  17q21.1(RARAx2)[100/100] | CCR | yes |
| *KMT2A*-r PDX #1 | m | 16 | 69.7 | 10.7 | 93 | no | no | NRAS mutation | 42~44,XY,t(6;11)(q27;q23)[cp2]/51,idem,+X,+der(6)t(6;11)(q27;q23),+8,+19,+21[5] | NR | no |
| *KMT2A*-r PDX #2 | f | 7 | 585 | 8.3 | 84 | no | yes | n.d. | 46,XX,t(9;11)(p22;q23)[8]/50,XX,idem,+3,+8,+18,+19[15] | CCR | no |
| *KMT2A*-r PDX #3 | m | 13 | 47.9 | 8.7 | 87 | no | no | n.d. | 47,XX,+8,t(9;11)(p22;q23) | ED | no |

**Supplemental Table 1.** Patient sample characteristics. WBC: white blood count, BM: bone marrow, CNS: central nervous system, SCT: stem cell transplantation, CCR: continuous complete response, NR: no response, ED: early death.

**References**

1. Rongvaux, A., et al., *Development and function of human innate immune cells in a humanized mouse model.* Nat Biotechnol, 2014. **32**(4): p. 364-72.

2. Pronk, C.J., et al., *Elucidation of the phenotypic, functional, and molecular topography of a myeloerythroid progenitor cell hierarchy.* Cell Stem Cell, 2007. **1**(4): p. 428-42.
